# Distinct non-contrast computerized tomography head findings in a patient with secondary polycythemia in rural Nepal: a captivating case report highlighting diagnostic challenges

**DOI:** 10.1097/MS9.0000000000001234

**Published:** 2023-09-01

**Authors:** Aastha Ghimire, Shailendra Katwal, Kusum Shrestha, Sunil Baniya

**Affiliations:** a Department of Radiology, Dadeldhura Subregional Hospital, Dadeldhura; b Patan Academy of Health Sciences, Lalitpur; c Kist Medical College and Teaching Hospital, KIST Hospital Road; d Nepalese Army Institute of Health Sciences, Kathmandu, Nepal

**Keywords:** case report, hyperdense vasculature, secondary polycythemia, tetralogy of fallot(TOF)

## Abstract

**Introduction:**

Tetralogy of Fallot is a cyanotic congenital heart disease which if untreated leads to death by adulthood. In rare cases who survive this can lead to secondary polycythemia due to chronic hypoxia who can present with multiple indistinct symptoms leading to diagnostic dilemma.

**Case presentation:**

The authors present a rare case of secondary polycythemia diagnosed in early fifties with the unique non-contrast computerized tomography head findings who presented with a spectrum of long standing symptoms and a history of neurological manifestations.

**Clinical discussion:**

Patients diagnosed with polycythemia in adulthood can have a background of an untreated congenital heart disease, which can be complemented further by appropriate radiological investigations in a resource poor setting.

**Conclusion:**

Polycythemia secondary to an untreated congenital heart disease in the age beyond 50 is a rare occurrence. The presentation of these patients might present clinicians with a diagnostic challenge which can be mitigated with an appropriate knowledge of the peculiar non-contrast computerized tomography head findings.

## Introduction

Highlights

- Tetralogy of Fallot is a cyanotic congenital heart disease which has a poor prognosis and low survival rates till adulthood if untreated.
- Congenital heart disease is one of the causes of secondary polycythemia, which can present with multiple vague symptoms along with neurological symptoms.
- Non -contrast computerized tomography head findings in polycythemia are very distinctive and can be a major tool to address the diagnostic uncertainty.

Polycythemia, also called erythrocytosis, refers to increased red blood cell mass, noted on laboratory evaluation as increased haemoglobin and haematocrit level^1^. True polycythemia can be stratified as primary and secondary polycythemia^1^. Cyanotic heart diseases with right-to-left shunt are one of the causes of secondary polycythemia; however, cases of uncorrected Tetralogy of Fallot (TOF) in an adult age are not very common^2^. Here, we present a rare case of a 52-year-old lady with polycythemia secondary to uncorrected TOF whose diagnosis was supplemented by the peculiar non-contrast computerized tomography (NCCT) head findings.

## Case details

A 52-year-old nonsmoker lady presented to the Outpatient department of our hospital in rural Nepal with complains of throbbing headache, palpitation and shortness of breath. She had been having these symptoms intermittently since her childhood but had an increase in severity for the last one month. These symptoms were accompanied by episodic transient blurring of vision, bluish discoloration of skin and dizziness. For the last 2 years she had also been having symptoms suggestive of peptic ulcer disease along with low mood and sleep disturbances. The patient was diagnosed with hypertension and was under treatment for the last 4 years. She reported an episode of weakness of right side of her body 15 years back which resolved without any residual symptoms and mentioned getting evaluated for some heart disease many years prior with no subsequent follow-up due to financial constraints. She had no history of daytime sleepiness, frequent coughing, noisy breathing and her partner confirmed that she did not snore or gasp in her sleep. She had no history of diabetes mellitus, hyperlipidemia, asthma, Chronic Obstructive Pulmonary Disease, renal disease and thyroid disease in self and family and was not under any medications apart from the non-diuretic antihypertensive agent. She had no recent significant weight changes. Assessment of her vital signs at presentation showed an oxygen saturation of 93%, heart rate of 115 beats per min, respiratory rate of 20 per min and a blood pressure of 120/90 mmHg. Examination findings were significant for clubbing and bilateral congested conjunctiva. Examination of nose, oral cavity, throat, chest and abdomen revealed no abnormalities. Her BMI was 22.5 kg/m^2^. On auscultation, first and second heart sounds were heard along with a pansystolic murmur.

**Figure 1:**
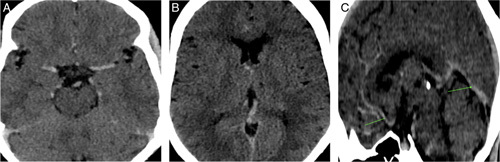
(A) Non-contrast axial computed tomography (CT) image at the level of midbrain shows the hyperdense bilateral middle cerebral arteries. (B) Non-contrast axial CT image at the level of basal ganglia shows the hyperdense bilateral internal cerebral veins (left>right). (C) Non-contrast midsagittal CT image showing hyperdense straight sinus, great cerebral vein (green arrow) and anterior cerebral artery (green line).

A complete blood count along with renal function test, liver function test, fasting blood sugar, chest X-ray and ECG were ordered. complete blood count showed haemoglobin and haematocrit levels of 21.2 g/dl and 69%, respectively. Chest X-ray revealed cardiomegaly but the bilateral lung fields were normal. No abnormalities were revealed in the rest of the investigations. A review of her old medical records was attempted which did not yield anything due to lack of electronic patient database. A meticulous search of old documents at home was requested to the patient. This unveiled an echocardiography done 15 years back reporting the findings of TOF. In the light of these findings and in accordance to her symptoms, a NCCT head was ordered, which revealed diffuse bilateral hyperdense vasculatures (Fig.1A, B, and C). She was then diagnosed with secondary polycythemia.

Phlebotomy sessions were scheduled and performed, which led to improvement of her symptoms and haematocrit levels. A non-diuretic antihypertensive agent was continued. The patient was counselled about her diagnosis, cause of her symptoms and was explained the need of surgical treatment which she was unable to comply with because of financial constraints.

She is currently under regular monthly follow-up in our hospital where she is assessed clinically and through regular haemoglobin and haematocrit level, her last haematocrit level being 42%. Her condition has been stable and static along with improvement of her mood. She has maintained a blood pressure of 120/80 mmHg and her symptoms of shortness of breath and headache have significantly improved.

## Discussion

Cyanotic congenital heart disease is one of the causes of physiologically appropriate secondary polycythemia^1^. However, uncorrected TOF survival is uncommon^2^. It has been reported that around 10% of affected persons can survive to adulthood, and only 5% reach 40 years of age as in our case^2^.

Clinical presentations of secondary polycythemia ranges from nonspecific symptoms like fatigue, headache, dizziness, pruritus and epigastric distress to vascular complications like hypertension, transient ischaemic attacks, arterial and venous thrombosis and major haemorrhage^1^. Many polycythemic patients are asymptomatic until late in disease^3^. In our case the patient was a 52-year-old lady who presented with a prolonged duration of multiple vague symptoms and a history of neurological symptoms. She was diagnosed with polycythemia secondary to a congenital heart disease based on an echocardiography report and after ruling out other causes of polycythemia by the aid of available investigations in the rural centre. NCCT head findings further provided important insights and supplemented this diagnosis.

Increased red blood cell mass in polycythemia can elevate blood viscosity, which can impair blood flow and make individuals susceptible to vaso-occlusive events^4,5^. This susceptibility makes radiological investigation in any patient diagnosed with secondary polycythemia presenting with nonspecific neurological symptoms like ours of utmost importance.

Electron density is the factor determining image contrast in CT studies^3^. In a normal adult with haematocrit ranging from 42 to 52%, the vessels appear isointense in non-contrast studies^3^. However, in patients with a haematocrit of more than 60%, the cerebral vessels may appear hyper dense even in non-contrast studies as a result of the increased red blood cell mass^3^. The hyper intensity is diffuse affecting Circle of Willis as well as dural sinuses^3^. These findings have been described very little in literatures which might mislead the diagnosis towards dural venous sinus thrombosis, which is also a possible complication of polycythemia^6^. So, it becomes vital to distinguish the peculiar findings of polycythemia itself appreciated in NCCT head from possible cerebrovascular event occurring in the background of the disease. The definite differentiation from dural venous sinus thrombosis requires further investigations like MRI head, CT venography or catheter venography^5^. However, clinical findings and adequate knowledge about the peculiar finding in the cerebral vessels of a person with polycythemia might be an important factor in making a correct diagnosis. Identification and discussion of cases like this would contribute further to the existing knowledge.

The treatment of secondary polycythemia is targeted towards treating the cause. So, the ideal treatment in this case would have been the surgical correction of the cardiac anomaly^1^. But since the patient could not undergo surgery, phlebotomy became the next best modality of treatment. The rationale behind repeated phlebotomies is that cytoreduction reduces hyper viscosity. Additionally, it induces a state of iron deficiency that would help retard red-cell proliferation^1^. Based on the CYTO-PV trial conducted in Italy a haematocrit of 45% is considered as target for the patients with secondary polycythemia in order to significantly lower the rates of cardiovascular deaths and thrombotic complications^7^. Weekly sessions of phlebotomy each removing 500 ml of blood is considered in practice in the patients who have a stable hemodynamics to meet the haematocrit target^1^. Other modalities of treatment include agents like hydroxyurea as a second line therapy to phlebotomy or an adjunctive therapy and Ruxolitinib, low dose aspirin and hypouricemic agents as adjuncts^8,9,10^. Hydroxyurea can be used with a standard daily dose of 500–1500 mg per day but the dosage has to be adjusted considering the platelet and neutrophil counts^1^. Ruxolitinib is a JAK2 inhibitor which can be used in patients intolerant or unresponsive to Hydroxyurea^9^. Studies also found that lower doses of aspirin could be safely used to reduce risk of developing thrombosis during the first three years of therapy^1,10^. Additional agents like antihistamines may be necessary for symptom control in patients who present with pruritus^11^. In addition to treatment of underlying cause, phlebotomy and medical management, counselling is an integral part of management of patients with polycythemia. Smoking cessation and a healthy diet should be encouraged. Moderate physical activity can be advised in patients whose mobility is not limited as it decreases the chances of thromboembolic complications^12^. Avoiding hot showers can be suggested in patients who have pruritus exacerbated by heat^12^. Genetic counselling and discussion with family should be promoted if the cause of polycythemia has a genetic component^1^. Symptomatic management of associated conditions is also a vital part of treatment. Assessment of the ongoing treatment modality, regular follow-up, evaluation of potential side effects of therapy and dose adjustments might be warranted. The importance of follow-up should be emphasized.

This patient suffered from the symptoms of an untreated congenital heart disease till the age of 52 years when she finally was appropriately diagnosed and treatment was discussed. Although her economic condition prevented her from getting a definitive treatment, other modalities of treatment (like phlebotomy in this case) offered significant change in the baseline condition of the patient.

## Conclusion

In a country like Nepal many patients in the rural settings suffer chronically with vague nonspecific symptoms without reaching a diagnosis. Constraints in healthcare services here are not only limited to poverty, inadequate machinery and equipment but also lack of adequate specialist services. There are many healthcare centres with adequate equipment with no professional manpower and vice versa. The diagnosis in this patient who was suffering from years was successfully made only because both these aspects were addressed at that particular setting. The availability of appropriate radiological equipment and competent specialist who could identify and distinguish the unique features in NCCT were the reasons that led her to a diagnosis and treatment in the rural setting minimizing the need for referral and cost to the patient. However, it is still important to address that more knowledge about conditions like this are to be described and disseminated along with the decentralization of specialist healthcare services.

## Ethical approval

Ethical approval is not required for case reports in my institution (Patan Academy of Health Sciences, Bagmati Lalitpur) so ethical approval was exempted.

## Consent

Written informed consent was obtained from the patient for the publication of this case report and accompanying images. A copy of written consent is available for review by the Editor in chief of this journal on request.

## Sources of funding

Not applicable.

## Author contribution

A.G.: conceptualization, mentor and reviewer for this case report and for data interpretation. S.K.: contributed in performing literature review and editing. S.B.: contributed in writing the paper and reviewer for the case. K.S.: contributed in writing the paper. All authors have read and approved the manuscript.

## Conflicts of interest disclosure

All the authors declare that they have no competing interest.

## Research registration unique identifying number (UIN)

Not applicable.

## Guarantor

Shailendra Katwal.

## Data availability statement

Not applicable.

## Provenence and peer review

Non commissioned, externally peer-reviewed.
